# Chronic IL9 and IL-13 Exposure Leads to an Altered Differentiation of Ciliated Cells in a Well-Differentiated Paediatric Bronchial Epithelial Cell Model

**DOI:** 10.1371/journal.pone.0061023

**Published:** 2013-05-09

**Authors:** Jeremy C. Parker, Surendran Thavagnanam, Grzegorz Skibinski, Jeremy Lyons, Jennifer Bell, Liam G. Heaney, Michael D. Shields

**Affiliations:** 1 Centre for Infection and Immunity, Queen's University Belfast, Belfast, Northern Ireland; 2 Royal Belfast Hospital for Sick Children, Belfast Health and Social Care Trust, Belfast, Northern Ireland; 3 The Royal Hospitals, Belfast Health and Social Care Trust, Belfast, Northern Ireland; 4 Paediatric Department, University Malaya Medical Centre, Kuala Lumpar, Malaysia; University of Liverpool, United Kingdom

## Abstract

Asthma is a chronic inflammatory disease characterised by airways remodelling. In mouse models IL-9 and IL-13 have been implicated in airways remodelling including mucus hypersecretion and goblet cell hyperplasia. Their role, especially that of IL-9, has been much less studied in authentic human *ex vivo* models of the bronchial epithelium from normal and asthmatic children. We assessed the effects of IL-9, IL-13 and an IL-9/IL-13 combination, during differentiation of bronchial epithelial cells from normal (n = 6) and asthmatic (n = 8) children. Cultures were analysed for morphological markers and factors associated with altered differentiation (MUC5AC, SPDEF and MMP-7). IL-9, IL-9/IL-13 combination and IL-13 stimulated bronchial epithelial cells from normal children had fewer ciliated cells [14.8% (SD 8.9), p = 0.048, 12.4 (SD 6.1), p = 0.016 and 7.3% (SD 6.6), p = 0.031] respectively compared with unstimulated [(21.4% (SD 9.6)]. IL-9 stimulation had no effect on goblet cell number in either group whereas IL-9/IL-13 combination and IL-13 significantly increased goblet cell number [24.8% (SD 8.8), p = 0.02), 32.9% (SD 8.6), p = 0.007] compared with unstimulated normal bronchial cells [(18.6% (SD 6.2)]. All stimulations increased MUC5AC mRNA in bronchial epithelial cells from normal children and increased MUC5AC mucin secretion. MMP-7 localisation was dysregulated in normal bronchial epithelium stimulated with Th2 cytokines which resembled the unstimulated bronchial epithelium of asthmatic children. All stimulations resulted in a significant reduction in transepithelial electrical resistance values over time suggesting a role in altered tight junction formation. We conclude that IL-9 does not increase goblet cell numbers in bronchial epithelial cell cultures from normal or asthmatic children. IL-9 and IL-13 alone and in combination, reduce ciliated cell numbers and transepithelial electrical resistance during differentiation of normal epithelium, which clinically could inhibit mucociliary clearance and drive an altered repair mechanism. This suggests an alternative role for IL-9 in airways remodelling and reaffirms IL-9 as a potential therapeutic target.

## Introduction

Asthma is a chronic inflammatory disease of the lower airways which tends to begin during childhood [1], [2]. One in seven children in the UK are affected by asthma and as a result represent a major financial burden on the NHS which is worsened when asthma is poorly controlled [3]. Additionally, asthma is one of the most common chronic diseases worldwide with approximately 300 million individuals affected [4]. Traditional treatments, including inhaled corticosteroids and short and long-acting β_2_-agonists, are used to control asthma symptoms and exacerbations, however in a small group of severe asthmatics their efficacy is poor. Additionally, current treatments do not address the underlying issue of airways remodelling.

Airways remodelling in asthma is characterised by non-reversible changes in the bronchial epithelium including goblet cell hyperplasia, mucus hyper-secretion, sub-epithelial fibrosis, smooth muscle hypertrophy and increased basement membrane thickening [5]. This in turn leads to airflow obstruction which can be life-threatening with approximately 1500 deaths per year in the UK as a result of asthma [6], [7]. The dramatic change in the phenotype of the airway is caused by a shift from a balanced Th1/Th2 phenotype to a chronic Th2 pro-inflammatory phenotype causing dysregulation and/or aberrant repair of the bronchial epithelium [8]–[10]. It has been reported that asthmatic airways have abnormal barrier function which in turn leads to chronic tissue injury and altered repair mechanisms [10], [11]. Several inflammatory mediators have been implicated in in the development of airways remodelling including the Th2 cell cytokines IL-9 and IL-13. Cytokines have become viable therapeutic targets because of the lack of effect of traditional therapies in severe asthma.

IL-9 is a pleiotropic Th2 cytokine released by a subset of CD4+ cells designated Th9 cells [12], [13] and has been identified as a candidate cytokine for asthma pathogenesis [14]. In murine models IL-9 stimulates mucin transcription and goblet cell hyperplasia [15] and by over-expressing IL-9 in the lungs of a transgenic mouse model it also induced IL-13 production from airway epithelial cells [16]. It is still unclear whether the role of IL-9 is that of a dominant cytokine or one of a helper cytokine in asthma. A recent study using cultures of micro-dissected murine terminal bronchioles has found that a combination of IL-9 and IL-13 increased goblet cell hyperplasia [17]. Xiang and colleagues suggested that IL-9 and IL-13 may act independently on airway epithelial cells to regulate mucin synthesis and in addition show an overall synergistic effect [17]. As a result of this and other studies, IL-9 and IL-13 have been identified as potential therapeutic targets. In particular, inhibition of IL-9 has shown promising results in reducing allergic inflammation and mucus hypersecretion in mice and in clinical studies an IL-9 blocking antibody has been shown to reduce exercise-induced asthma [18].

In the only study to date to have examined differentiated human airway epithelial cells, Vermeer et al. found that IL-9 induced goblet cell hyperplasia both during differentiation and in a mechanical injury model using cells obtained from the trachea [19]; this effect was not seen in cells exposed to IL-9 post-differentiation which suggested that this effect only occured during the differentiation process. The role of IL-9 in bronchial epithelial cell differentiation, how it affects cells from asthmatic subjects and how it acts in combination with IL-13 in paediatric bronchial epithelial cell differentiation remains unclear. With the potential of IL-9 being a clinical therapeutic target, it is important to understand its actions on the bronchial epithelium, in both health and disease. The *ex vivo* well-differentiated model of bronchial epithelial cells has been shown to closely resemble the *in vivo* epithelium making it the ideal model for further study of the effects of the Th2 cytokines on airways remodelling [20].

In this paper we will assess morphological markers of airways remodelling as an output of altered differentiation in response to direct stimulation of the bronchial epithelial cells with IL-9 and IL-13 alone and combined. These will include quantification of goblet and ciliated cell number, MUC5AC, the major mucus forming mucin, SAM-pointed-domain-containing-Ets-like factor (SPDEF), a transcription factor known to be involved in the goblet cell hyperplasia pathway [21] and matrix metalloproteinase (MMP) -7 which has been shown to have dysregulated expression in the asthmatic epithelium due to Th2 cytokine exposure [22].

We hypothesise that IL-9 alone and in combination with IL-13 in a synergistic fashion will contribute to goblet cell hyperplasia and a reduction in ciliated cells in paediatric bronchial epithelial cell differentiation. We used a well-differentiated model of bronchial epithelial cells from normal (PBEC(N)) and asthmatic (PBEC(A)) children.

## Materials and Methods

### Ethics statement

Written informed parental consent and where appropriate child assent was obtained. This study was approved by the Office of Research Ethics Committees Northern Ireland (ORECNI, Reference Number: 07/NIR02/141).

### Subjects

Children less than 12 years (mean age 7.3 years [range: 1 to 12 years]) attending elective surgical procedures at the Royal Belfast Hospital for Sick Children were recruited. A doctor administered questionnaire was used to record the clinical history. Recruited children included asthmatics (recurrent wheezing within the last year – mean age 6.3 years, n = 8) or normal controls (mean age 8.5 years, n = 6) who had never wheezed. Seven of the asthmatic children had other atopic features (allergic rhinitis and/or eczema, or an elevated total IgE). All asthmatic children were being treated with inhaled corticosteroids (ICS) and four were also on long-acting beta 2 agonists (two of these additionally were on leukotriene antagonists). The healthy controls had no respiratory symptoms and were on no asthma therapy. One healthy control child had eczema and this and one other child had an isolated elevated total IgE.

### Isolation of primary PBECs

Non-bronchoscopic bronchial brushings were used to sample and culture bronchial epithelial cells from asthmatic children (n = 8) and normal children (n = 6) as previously described [23], [24]. A number of these samples were also used in our previous study for IL-13 exposure [25]. This represents a culture success rate of 70% of PBEC samples received. All brush washings were analysed for viruses using a multi-viral PCR analysis and only uncontaminated cultures were used [26].

### ALI cultures for establishment of well-differentiated mucociliary epithelium

Air liquid interface (ALI) cultures were grown in line with methods previously described [23], [24], [25], [27], [28]. Briefly, all cells from subjects used in this study were grown at ALI at passage 2 in ALI medium consisting of a 50∶50 mixture of AEGM (Promocell, Heidelberg, Germany) and DMEM (Invitrogen Ltd, Paisley, UK) supplemented with bovine pituitary extract (52 µg/ml), epidermal growth factor (0.5 ng/ml), insulin (5 µg/ml), hydrocortisone (0.5 µg/ml), epinephrine (0.5 µg/ml), transferrin (10 µg/ml), bovine serum albumin (1.5 µg/ml), penicillin/streptomycin (100 IU/ml/100 µg/ml) and retinoic acid (50 nM). The cells were grown in transwells submerged for the first 9 to 14 days, during which time the culture medium was changed on day 1 and every other day thereafter. Once the cells reached 100% confluence, ALI was created by removing the apical medium and restricting the culture feeding to the basolateral compartment. Following ALI creation, the culture medium was changed on alternate days and the cells were then differentiated over 28 days to ensure full differentiation as assessed by the presence of beating cilia and mucus on the apical surface of the cultures. Primary paediatric bronchial epithelial cells (PBECs) were fed every other day with ALI medium alone as an unstimulated control, or supplemented with 20 ng/ml IL-9, 20 ng/ml IL-13 or 20 ng/ml IL-9/IL-13 combination during the differentiation process (PeProTech EC, Scotland, UK). These concentrations were selected following intial dose-response studies (data not shown) in the case of IL-13. All WD-PBECs included reached full differentiation at ALI.

### Transepithelial Electrical Resistance (TEER) measurements

TEER was measured on days 7, 14, 21, and 28 to ensure the formation and integrity of tight junctions between cells in the epithelium using an EVOM meter (World Precision Instruments, FL, USA).

### ELISA measuring IL-9 and IL-13

Cytokine concentrations were measured from aliquots of basolateral culture medium and apical washings taken on days 7, 14, 21 and 28 of culture using commercial ELISA kits (IL-9: PeProTech EC, Scotland, UK; IL-13: Pierce BioScience, Northumberland, UK) as per manufacturers instructions.

### Immunocytochemistry (ICC) for goblet and ciliated cell markers

ICC was carried out using methods previously described [24], [25]. Briefly, immuno-staining was performed using mouse anti-MUC5AC antibody (1∶100) (Abcam, UK) and mouse anti-acetylated alpha tubulin antibody (1∶700) (Abcam, UK) for goblet and ciliated cells respectively. The reaction between the antigen and the antibody was detected using a perixodase-conjugated anti-mouse secondary antibody (Vector Laboratories, Peterborough, UK). The slides were then stained with DAB substrate kit (Vector Laboratories, Peterborough, UK) for visualization. Negative controls were subjected to routine conditions with the omission of the primary antibody. MUC5AC (goblet cells) and acetylated alpha tubulin (ciliated cells) positive cells were counted at a magnification of ×40 blindly in 6 fields of vision. A total number of 500 cells per slide were enumerated and the number of positive cells for MUC5AC or acetylated alpha tubulin was then expressed as a percentage of the total cells counted per slide. Three slides per stain (MUC5AC or acetylated alpha tubulin) per insert were enumerated and in this paper the results represent the mean percentage of those three slides. As there were no significant differences between the total numbers of cells at the end of the culture period the percentages presented are representative of the total proportions of goblet and ciliated cells within the epithelium.

### Immunofluorescence (IF) and confocal microscopy

IF and confocal microscopy were carried out using methods previously described [24], [25]. Briefly, a 1∶100 dilution of mouse monoclonal antibody against acetylated alpha tubulin (ciliated cells) (Abcam, Cambridge, UK), a 1∶100 dilution of mouse monoclonal antibody against cytokeratin 5/8 conjugated to AF488 (Insight Biotechnology, UK) or a 1∶100 dilution of rabbit polyclonal antibody against MMP-7 (Abcam, UK) was incubated with selected inserts overnight at 4°C. Cells were exposed to a 1∶250 dilution of goat anti-rabbit IgG Alexafluor 568 (MMP-7) (Invitrogen Ltd, Paisley, UK) or a 1∶250 dilution of Alexafluor 488 goat anti-rabbit IgG (ciliated cells) (Invitrogen Ltd, Paisley, UK) at 4°C in the dark for 1 hour. The membrane was cut out from the insert using a scalpel and then mounted on a slide using Vectashield with DAPI (Vector Laboratories, Peterborough, UK). Fluorescent images were viewed on a Leica SP5 confocal DMI 6000 inverted microscope equipped with a krypton-argon laser as the source for the ion beam using ×40 oil immersion (numerical aperture 1.25). Images were captured and viewed using LAS AF (Leica) acquisition software. Negative controls (omission of primary antibody) were used to test the specificity of the antibodies and the staining protocol.

Z-series stacks of representative cultures acquired using the Leica SP5 confocal microscope were analysed step-wise for total staining area of MMP-7 using FIJI software (Image J) to detect semi-quantitatively the localisation (apical, middle or basal) of MMP-7 throughout the PBEC(N) & (A) cultures.

### RNA extraction and Real-time PCR

RNA extraction was carried out using RNeasy Mini kit (Qiagen, Crawley, UK) according to the manufacturer's instructions and quantified on a spectrophotometer. First Strand cDNA Synthesis Kit for RT-PCR (AMV) (Roche, UK) was then used according to the manufacturer's protocol. For the real time PCR reaction, the DNA amplification was carried out using the Fast Start Universal SYBR Green Master (Rox) (Roche, UK) according to the manufacturers' protocol. For each PCR reaction separate PCR Master Mixes were prepared containing GAPDH (Tebu-bio, Peterborough, UK), IL-13 (R&D Systems, Abingdon, UK), MUC5AC (Invitrogen, Paisley, UK), MMP-7 (Invitrogen, Paisley, UK) and SPDEF (Invitrogen, Paisley, UK) primers. MUC5AC, MMP-7 and SPDEF primer sequences are listed in the supplementary section (Primer Sequence S1). A final total volume of 10 µl was constituted with the addition of 1 µl of cDNA added into each Master Mix. Samples were placed in individual wells in a Thermo-Fast 96-well detection plate and run on the spectrofluorometric thermal cycler AB 7000 (ABI Prism).

### ELISA measuring MUC5AC mucin

MUC5AC mucin was measured from apical washings taken from the cultures on day 28 using ELISA. The method used was adapted from Takeyama and co-workers [29]. Results were expressed as optical density proportional to MUC5AC secretion, which is directly proportional to the amount of mucin present, with a positive mucin control used across experiments.

### Statistical analysis

Our *a priori* aims in this study were to make planned comparisons between the effects of IL9, alone and combined with IL13 versus no stimulation (control) on differentiation and to compare IL9 with IL13 stimulation. We first compared stimulation responses using one way repeated measures ANOVA (RM-ANOVA) and if the overall RM-ANOVA was statistically significant (p≤0.05) we then performed planned unadjusted paired t-tests expressing results as mean (standard deviation, SD). For comparisons of TEER measurements we analysed data using a two-way repeated measures ANOVA (TEER over Time by Normal versus Asthmatic).

## Results

All cultures exhibited a well-differentiated pseudostratified epithelium with ciliated cells and goblet cells as well as TEER values above 300 ohm.cm^2^, which supported the visual appearance of a morphologically intact epithelium with tight junction formation. No IL-13 mRNA was detected in unstimulated cells or cells exposed to IL-9 and IL-9/IL-13 combination using real time PCR (data not shown). Neither IL-9 or IL-13 were detected in basolateral supernatants or apical washings using ELISAs (data not shown). The total cell counts at the end of the culture period showed there to be no significant difference between unstimulated and IL-9, IL-13 or IL-9/IL-13 combination stimulated cultures in both groups (data not shown). It is worth noting that it is extremely unlikely given the number of passages the cells go through (cells are differentiated at passage 2) along with the length of time from sampling to completion of culture (7–10 weeks) that there would be any carry-over effect of inhaled corticosteriods.

### PBEC(N) stimulation with IL-9, IL-13 or IL-9/IL-13 combination

**Figure 1:**
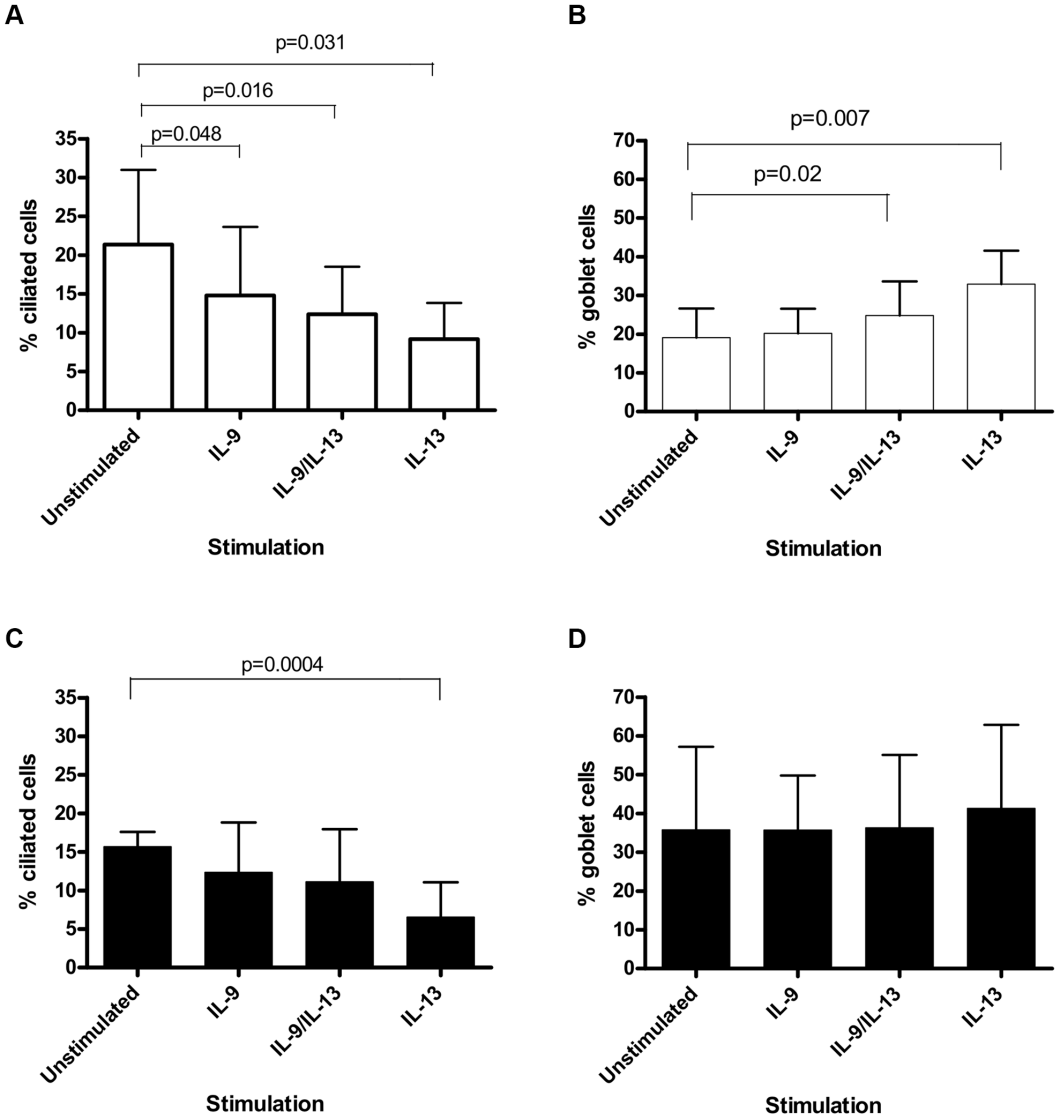
Percentage of goblet and ciliated cells in PBEC(N) and (A) cultures. (A) Numbers of ciliated cells expressed as a percentage of total on day 28 for PBEC(N) stimulated with IL-9, IL-9/IL-13 combination and IL-13. Values are expressed as mean (error bar represents 1 SD). A repeated measures ANOVA overall p value of p = 0.05 was obtained. The percentage of ciliated cells decreased with IL-9 (p = 0.048), IL-9/IL-13 combination (p = 0.016) and IL-13 (p = 0.031) stimulations. (B) Numbers of goblet cells expressed as a percentage of total on day 28 for PBEC(N) stimulated with IL-9, IL-9/IL-13 combination and IL-13. Values are expressed as mean (error bar represents 1 SD). A repeated measures ANOVA overall p value of p = 0.000007 was obtained. The percentage of goblet cells increased with IL-9/IL-13 combination stimulation (p = 0.02) and IL-13 stimulation (p = 0.007). (C) Numbers of ciliated cells expressed as a percentage of total on day 28 for PBEC(A) stimulated with IL-9, IL-9/IL-13 combination and IL-13. Values are expressed as mean (error bar represents 1 SD). A repeated measures ANOVA overall p value of p = 0.011 was obtained. The percentage of ciliated cells decreased with IL-13 stimulation (p = 0.0004). (D) Numbers of goblet cells expressed as a percentage of total on day 28 for PBEC(A) stimulated with IL-9, IL-9/IL-13 combination and IL-13. Values are expressed as mean (error bar represents 1 SD). A repeated measures ANOVA overall p value of p = 0.15 was obtained. There was no statistically significant increase in the percentage of goblet cells under any cytokine stimulation.

Regarding ciliated cells we found that all cytokine stimulations caused a significant reduction in the percentage of ciliated cells [IL-9 stimulated: mean 14.8% (SD 8.9), p = 0.048); IL-9/IL-13 combination: mean 12.4% (SD 6.1), p = 0.016; IL-13 stimulated: mean 7.3% (SD 6.6), p = 0.031)], versus unstimulated [unstimulated: mean 21.4% (SD 9.6)] (Figure 1A). No difference was seen in the percentage of goblet cells in PBEC(N) after stimulation with IL-9 alone [unstimulated: mean 18.6% (SD 6.2); IL-9 stimulated: mean 20.2% (SD 6.3)] (Figure 1B). IL-9/IL-13 combination and IL-13 alone significantly increased the percentage of goblet cells compared with unstimulated control [IL-9/IL-13 combination: mean 24.8% (SD 8.8), p = 0.02; IL-13 stimulated: mean 32.9% (SD 8.6), p = 0.007; versus unstimulated: mean 18.6% (SD 6.2) (Figure 1B)].

**Figure 2:**
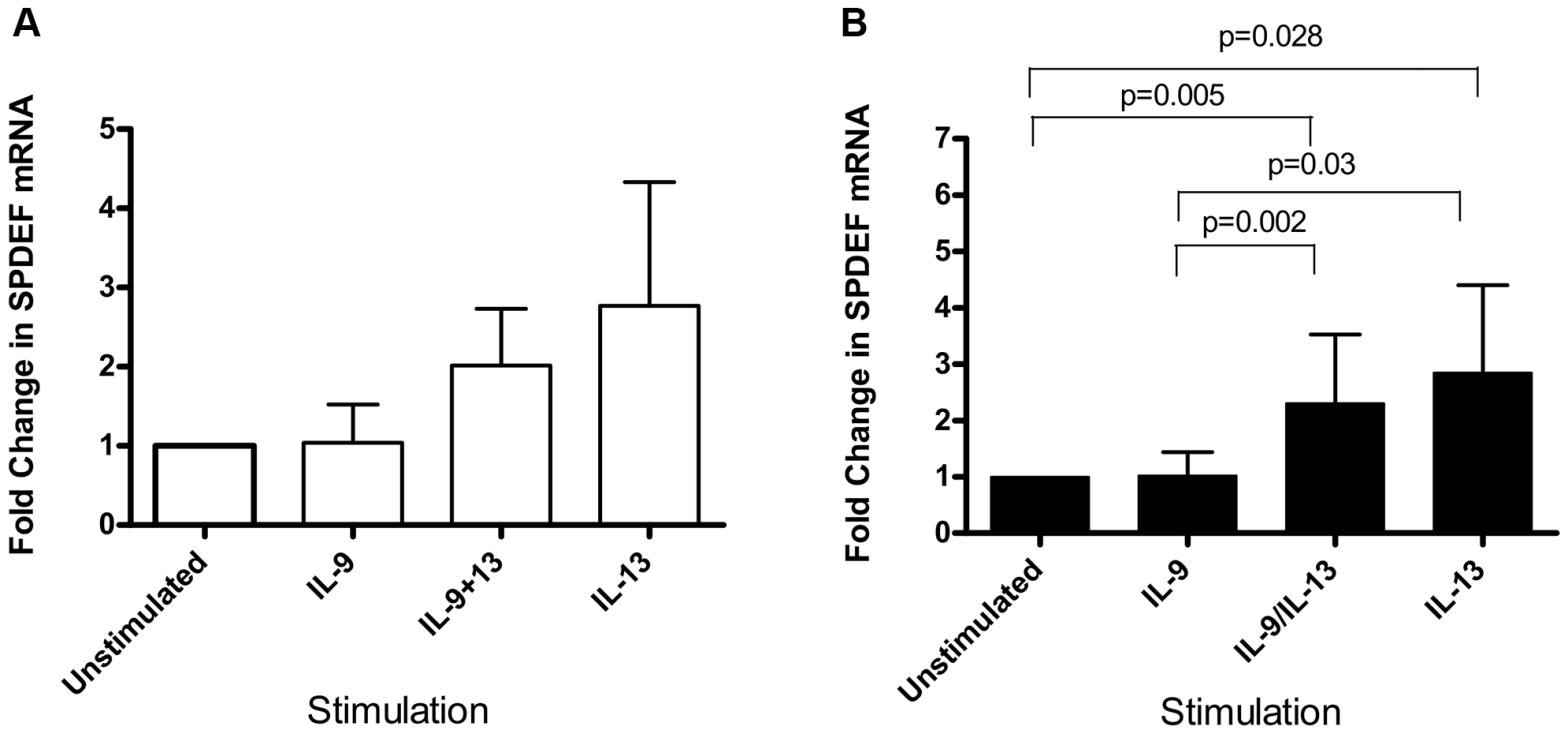
Fold change of SPDEF mRNA measured using real time PCR. (A) In PBEC(N) on day 28 of ALI culture we observed an increase in SPDEF mRNA by stimulation with IL-9/IL13 combination and IL-13 however this was not statistically significant. A repeated measures ANOVA overall p value of p = 0.11 was obtained. (B) In PBEC(A) a repeated measures ANOVA overall p value of p = 0.0004 was obtained. A significant difference was observed only with IL-9/IL-13 combination (p = 0.005) and IL-13 stimulation (p = 0.028) compared with unstimulated. Additionally significant increases were also observed between IL-9 and IL-9/IL-13 combination (p = 0.002) and between IL-9 and IL-13 (p = 0.03).

IL-9 stimulation did not increase SPDEF mRNA compared with control [IL-9 stimulation: mean 1.038 (SD 0.49)] whereas IL-9/IL-13 combination and IL-13 did but the increase was not statistically significant [IL-9/IL-13 combination: mean 2.02 (SD 0.72); IL-13: mean 2.77 (SD 1.6)] (Figure 2A).

**Figure 3:**
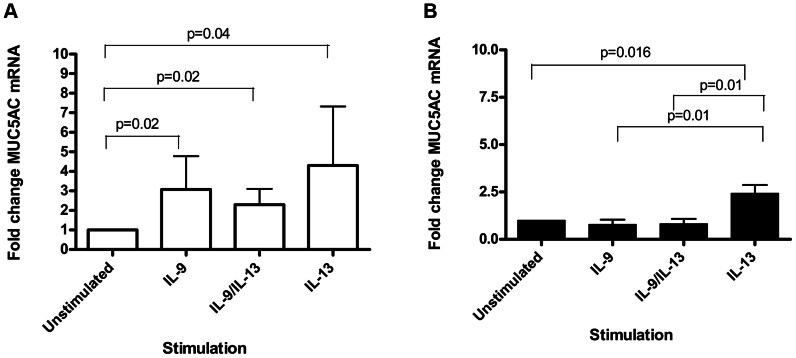
Fold change of MUC5AC mRNA measured using real time PCR. (A) In PBEC(N) on day 28 of ALI culture we observed a repeated measures ANOVA overall p value of p = 0.05. A significant increase in MUC5AC mRNA by stimulation with IL-9 (p = 0.02), IL-9/IL13 combination (p = 0.02) and IL-13 (p = 0.04) was observed. (B) In PBEC(A) a repeated measures ANOVA overall p value of p = 0.00098 was obtained. A significant increase compared with unstimulated was observed only with IL-13 stimulation (p = 0.016). Additionally, there were significant differences in MUC5AC mRNA between IL-9 and IL-13 stimulations (p = 0.01) and between IL-9/IL-13 combination and IL-13 stimulations (p = 0.01).

MUC5AC mRNA increased (fold change) significantly in PBEC(N) for all cytokine stimulations [IL-9 stimulated: mean 3.1 (SD 1.7), p = 0.02; IL-9/IL-13 combination: mean 2.3 (SD 0.8), p = 0.02; IL-13 stimulation: mean 4.3 (SD 3.0), p = 0.04] (Figure 3A).

**Figure 4:**
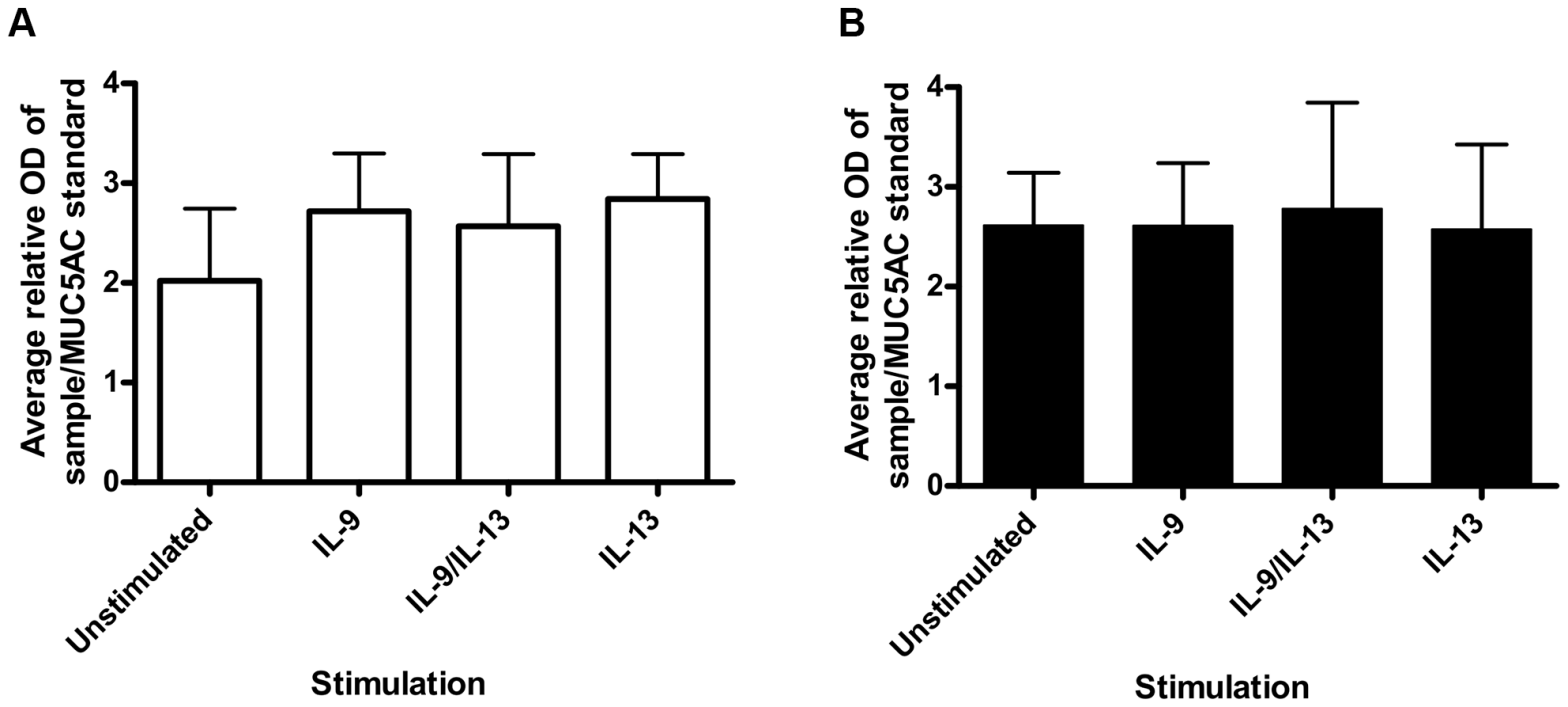
Average relative optical density of apical washings measured for MUC5AC secretion. Values expressed are mean (error bar represents 1 SD). (A) In PBEC(N) on day 28 of ALI culture we observed higher amounts of MUC5AC at all cytokine stimulations, however this was not statistically significant. A repeated measures ANOVA overall p value of p = 0.068 was obtained. (B) In PBEC(A) we observed no significant difference in the amount of MUC5AC secreted in any of the cytokine stimulation groups. A repeated measures ANOVA overall p value of p = 0.8 was obtained.

A trend for increased secreted MUC5AC mucin at the apical surface was observed using ELISA between unstimulated [unstimulated: mean 2.02 (SD 0.7)] and all cytokine stimulations [IL-9 stimulated: mean 2.72 (SD 0.6); IL-9/IL-13 combination: mean 2.6 (SD 0.7); IL-13 stimulated: mean 2.84 (SD 0.4)] (Figure 4A).

**Figure 5:**
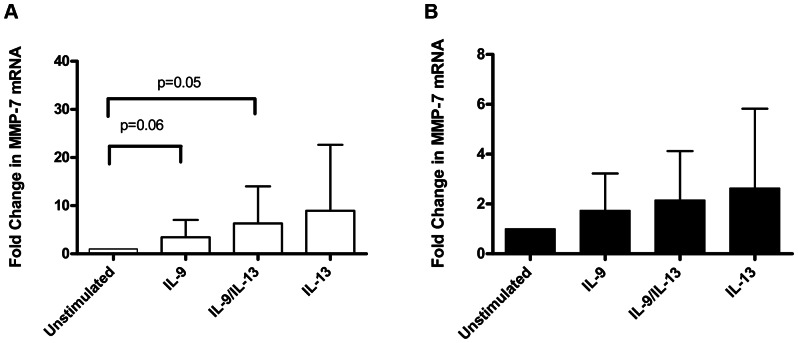
Fold change of MMP-7 mRNA measured using real time PCR. (A) In PBEC(N) on day 28 of ALI culture we observed a repeated measures ANOVA overall p value of p = 0.04. IL-9/IL13 combination (p = 0.05) and IL-9 stimulation showed an increase in MMP-7 mRNA (p = 0.06) as did IL-13 however these were not significant. (B) In PBEC(A) a there was no significant increase with any stimulation. A repeated measures ANOVA overall p value of p = 0.81 was obtained.

MMP-7 mRNA increased (fold change) in PBEC(N) for IL-9/IL-13 combination stimulation [IL-9/IL-13 combination: mean 6.3 (SD 7.7), p = 0.050] but not significantly. IL-9 and IL-13 stimulation [IL-9 stimulated: mean 3.4 (SD 3.6), p = 0.06; IL-13 stimulation: mean 9.0 (SD 13.7)] showed a similar trend to that of the IL-9/IL-13 combination (Figure 5A).

**Figure 6:**
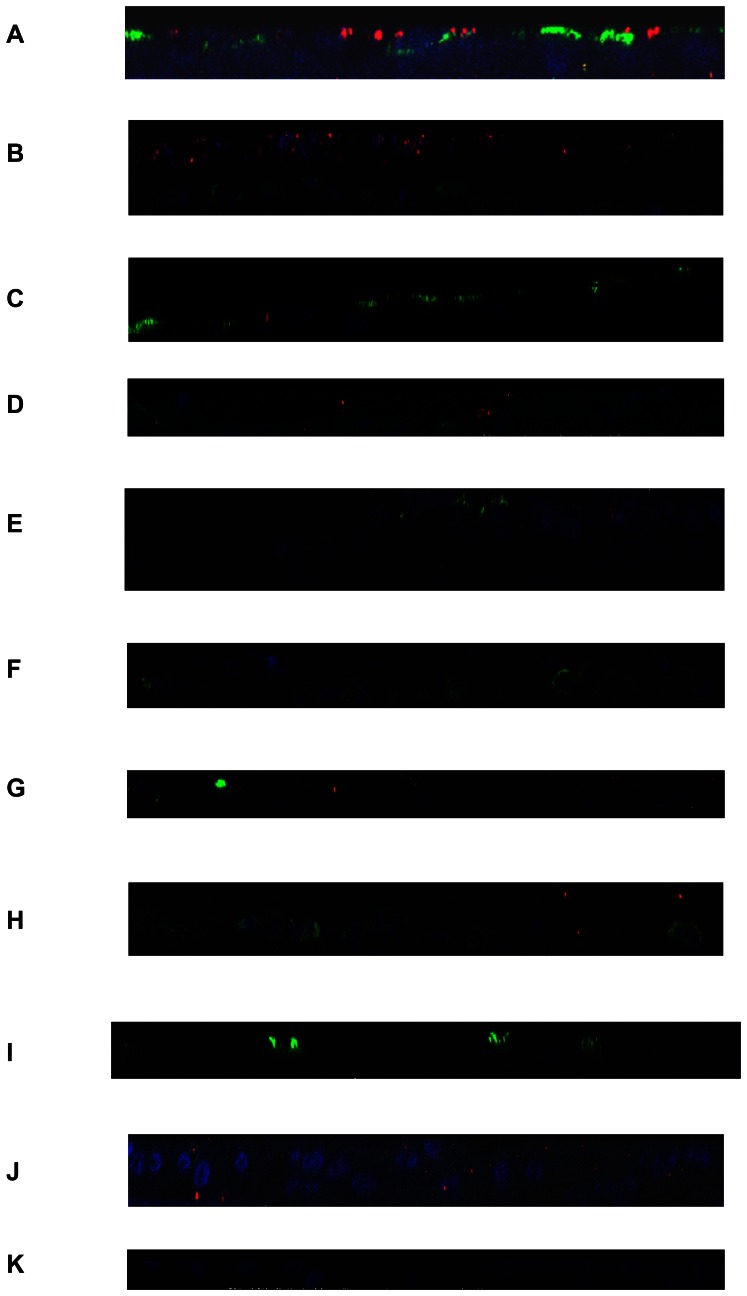
Immunofluorescent staining coupled with confocal microscopy for assessment of localisation of MMP-7 in representative PBEC(N) and (A). Representative z-stack orthogonal cut images of MMP-7 (red) and ciliated cell (green) expression of PBEC(N) at day 28 ALI culture for unstimulated (A), IL-9 stimulated (C), IL-9/IL-13 combination stimulated (E), IL-13 stimulated (G) and representative z-stack orthogonal cut images of MMP-7 (red) and basal epithelial cells (green) expression of PBEC(N) at day 28 ALI culture for unstimulated (B), IL-9 stimulated (D), IL-9/IL-13 combination stimulated (F), IL-13 stimulated (H). PBEC(A) representative images of MMP-7 (red) and ciliated cells (green) for unstimulated (I) and MMP-7 (red) and basal epithelial cells (green) for unstimulated (J). Negative control (K). The nuclei in each image are stained with DAPI (blue).

**Figure 7:**
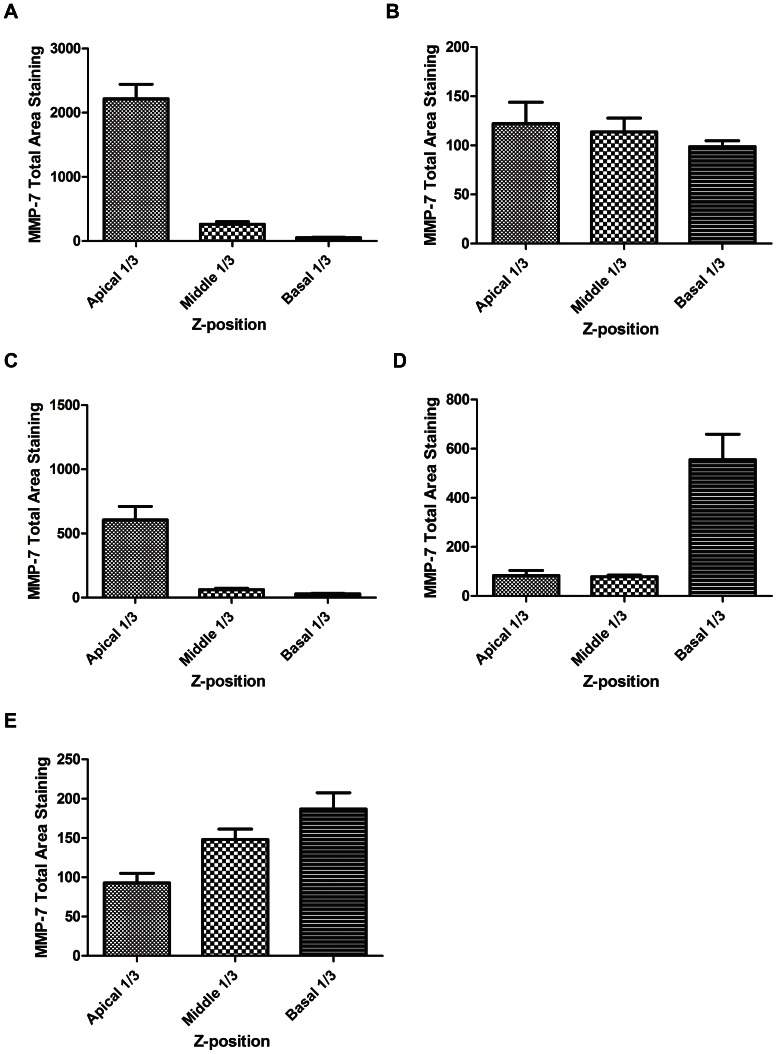
Semi-quantification of Total Area Staining for MMP-7 localisation throughout Z-series stacks of representative PBEC(N) and (A). (A) Unstimulated PBEC(N) on Day 28 of culture demonstrates high levels of staining for MMP-7 localisation in the Apical layer with minimal staining in the middle and basal layers. (B) IL-9 stimulated PBEC(N) on Day 28 of culture demonstrates a more even spread of MMP-7 staining throughout all levels of the culture. (C) IL-9/IL-13 combination stimulated PBEC(N) on Day 28 of culture demonstrates a higher expression pattern of MMP-7 in the Apical layers with lower staining in the middle and basal layers of the culture. (D) IL-13 stimulated PBEC(N) on Day 28 of culture demonstrates a low apical and middle expression of MMP-7 and a higher basal expression in the culture. (E) Unstimulated PBEC(A) on Day 28 of culture demonstrates a progressive increase in MMP-7 expression from the apical to the basal layers of the culture.

In terms of MMP-7 localisation within the epithelial cells we found that in unstimulated epithelium, MMP-7 expression (red) was localised mainly to the apical surface along with the ciliated cells (green) (Figure 6A) and separate from the basal cells (green) of the epithelium (Figure 6B & 7A). When stimulated with IL-9 we found dysregulation of MMP-7 (red) at the apical surface (Figure 6C) while being more dispersed throughout the middle and basal epithelial layers (green) (Figure 6D & 7B). When stimulated with IL-9/IL-13 combination we noticed a similar localisation of MMP-7 (red) expression as with the control cultures at the apical surface, albeit with fewer ciliated cells (green) (Figure 6E) and stronger basal cell (green) staining (Figure 6F &7C) with lower expression of MMP-7 (red) expressed throughout. IL-13 stimulation not only decreased ciliated cell number (green) and reduced apical MMP-7 (red) expression (Figure 6G) but was also associated with increased MMP-7 expression (red) throughout the basal epithelial cell layers (green) (Figure 6H & 7D). Negative controls were also analysed for non-specific staining (Figure 6K).

### PBEC(A) stimulation with IL-9, IL-13 or IL-9/IL-13 combination

In relation to ciliated cell numbers, we found that only IL-13 stimulation alone contributed to a significant reduction in the percentage of ciliated cells [IL-13 stimulation: mean 5.8% (SD 1.6)] compared with unstimulated [unstimulated: mean 15.5% (SD 2.3), p = 0.0004)] (Figure 1C). This reduction in the percentage of ciliated cell numbers was not seen in asthmatic cells with IL-9 stimulation alone or in combination with IL-13 (Figure 1C). The percentage of goblet cells in PBEC(A) increased following stimulation with IL-13 [IL-13 stimulation: mean 45.2% (SD 17.2)] compared with unstimulated [unstimulated: mean 35.8% (SD 21.4)] (Figure 1D), however this was not statistically significant. No differences were seen with IL-9 stimulation alone or in combination with IL-13 (Figure 1D).

SPDEF mRNA showed a significant increase under IL-9/IL-13 and IL-13 stimulation compared with control [IL-9/IL-13 combination: mean 2.3 (SD 1.2), p = 0.005; IL-13 stimulation: mean 2.84 (SD 1.5), p = 0.028] (Figure 2B). Additionally SPDEF mRNA also showed significant differences between IL-9 and IL-9/13 combination [IL-9 stimulation: mean 1.02 (SD 0.4); IL-9/IL-13 combination: mean 2.3 (SD 1.2), p = 0.002] and IL-13 [IL-9 stimulation: mean 1.02 (SD 0.4); IL-13 stimulation: mean 2.84 (SD 1.5), p = 0.03] (Figure 2B).

MUC5AC mRNA from PBEC(A) showed a significant fold change only in IL-13 stimulated PBEC(A) compared with unstimulated control [IL-13 stimulation: mean 2.43 (SD 1.2), p = 0.016] (Figure 3B). Additionally IL-13 showed a significant increase in MUC5AC mRNA compared with IL-9 alone [IL-9 stimulation: mean 0.79 (SD 0.6), p = 0.01] and IL-9/IL-13 combination [IL-9/IL-13 combination: mean 0.82 (SD 0.7), p = 0.01] (Figure 3B). In PBEC(A) no difference was observed in secreted MUC5AC following any of the cytokine stimulations [IL-9 stimulated: mean 2.6 (SD 0.6); IL-9/IL-13 combination: mean 2.8 (SD 1.1); IL-13 stimulated: mean 2.6 (SD 0.9)] when compared with unstimulated control [unstimulated: mean 2.6 (SD 0.5)] (Fig. 4B).

MMP-7 mRNA showed no significant increase (fold change) in PBEC(A) for any of the stimulations [IL-9 stimulated: mean 1.7 (SD 1.4); IL-9/IL-13 combination: mean 2.2 (SD 2.0); IL-13 stimulation: mean 2.6 (SD 3.2)] however all stimulations showed a trend for increase (Figure 5B).

In terms of MMP-7 staining, the asthmatic epithelium showed dysregulated expression of MMP-7 (red) along with fewer ciliated cells (green) at the apical surface (Figure 6I) and a large proportion of basal undifferentiated epithelial cells (green) with greater MMP-7 (red) expression present throughout the middle and lower epithelial cell layers (Figure 6J & 7E).

### Comparison of TEER values between PBEC(N) and PBEC(A) stimulated with IL-9, IL-9/IL-13 combination and IL-13

**Figure 8:**
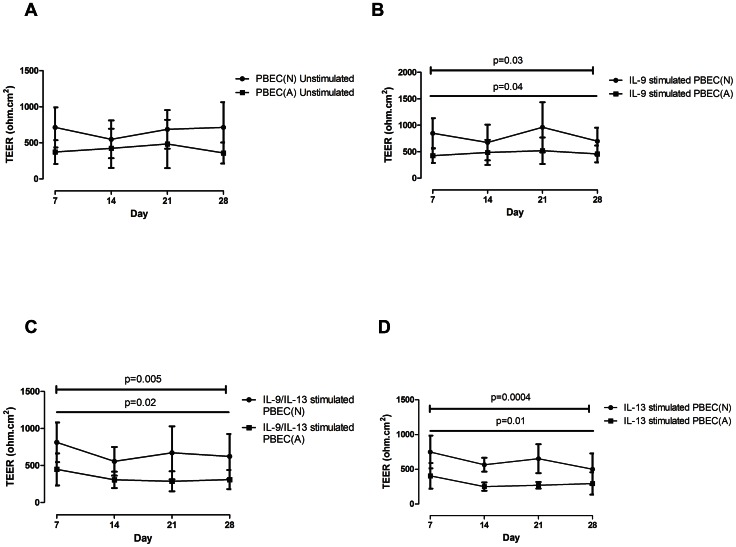
TEER values of PBEC(N) and PBEC(A) under stimulation with IL-9, IL-9/IL-13 combination and IL-13. Values are expressed as mean (error bar represents 1 SD) (straight line depicts analysis over Time; barred line depicts analysis between Clinical (Normal versus Asthmatic)). (A) TEER values between PBEC(N) and PBEC(A) displayed no significant differences under unstimulated conditions over time, between clinical phenotype (Normal versus Asthmatic) or by interaction (ie/. does PBEC(A) behave differently from PBEC(N) over time). (B) TEER values under stimulation with IL-9 demonstrated a significant reduction between PBEC(N) and PBEC(A) over time (Time p = 0.04) and between clinical (Clinical p = 0.03), however not by interaction (Interaction p = 0.09). (C) TEER values under stimulation with IL-9/IL-13 combination demonstrated a significant reduction between PBEC(N) and PBEC(A) over time (Time p = 0.02) and between clinical (Clinical p = 0.005), however not by interaction (Interaction p = 0.7). (D) TEER values under stimulation with IL-13 demonstrated a significant reduction between PBEC(N) and PBEC(A) over time (Time p = 0.01) and between clinical (Clinical p = 0.0004), however not by interaction (Interaction p = 0.4).

TEER values compared over time by clinical description (Normal or Asthmatic) using a 2-way repeated measures ANOVA demonstrated no significant difference between PBEC(N) and PBEC(A) in any of the outcomes measured under unstimulated conditions (time p = 0.3; clinical p = 0.07; interaction (ie. does PBEC(A) behave differently from (PBEC(N) over time) p = 0.07) (Figure 8A). Under stimulation with IL-9 there were significant differences over time and for clinical (time p = 0.04; clinical p = 0.03) however there was no significant interaction (interaction p = 0.09) (ie/. PBEC(N) and PBEC(A) respond in a similar way to stimulation with IL-9) (Figure 8B). Similar results were observed with IL-9/IL-13 stimulation (time p = 0.02; clinical p = 0.005; interaction p = 0.7) (Figure 8C) and with IL-13 (time p = 0.01; clinical p = 0.0004; interaction p = 0.4) (Figure 8D).

## Discussion

Our main findings from this study were (1) that IL-9, IL-9/IL-13 combination and IL-13 stimulation, in PBEC(N), and IL-13 in PBEC(A), resulted in a significant reduction in the ciliated cell number; (2) that IL-9 stimulation alone did not induce goblet cell hyperplasia in either PBEC(N) or PBEC(A); (3) IL-9 showed no synergy with IL-13 stimulation with respect to goblet cell hyperplasia; (4) IL-9, IL-9/IL-13 combination and IL-13 stimulation in PBEC(N) and PBEC(A) significantly reduced TEER over the duration of the differentiation process.

Although IL-9 has been implicated in goblet cell hyperplasia [19] we have found that it had no significant effect on goblet cell number in differentiating PBEC(N) and PBEC(A). This data differs, in part, with that of Vermeer and colleagues who demonstrated that IL-9 induced goblet cell hyperplasia during differentiation or in the already differentiated epithelium following an epithelial insult [19]. There were a few notable differences between the two studies. Vermeer studied adult tracheal airway cells whereas we used paediatric bronchial epithelial cells. Additionally, Vermeer and colleagues used a higher concentration of IL-9 and IL-13 for their experiments. We have previously shown that 20 ng/ml IL-13 alone induces a significant increase in goblet cell numbers [25] and this is an observation we have again confirmed in this study, thereby demonstrating consistency in both our model and the culture conditions. This may go some way to explaining some of the differences observed. In combination with IL-9, the IL-13 effect on goblet cell numbers is observed; however we found no evidence for synergy between IL-9 and IL-13, as suggested by Xiang and colleagues [17]. Instead, our data suggests that IL-13 is acting alone and, if anything, when combined with IL-9, this tended to reduce the effect of IL-13. This may suggest an inhibitory effect of IL-9 on the function of IL-13 which certainly warrants future investigation.

We observed an up-regulation of MUC5AC mRNA in PBEC(N), with a subsequent trend to increased MUC5AC secretion in apical supernatants, suggesting that with further investigation, IL-9 may have a role to play in mucin production. While goblet cell hyperplasia and increased mucus secretion are characteristics of the asthmatic epithelium they are separate morbidities. This role for IL-9 would fit with the observation of reduced mucus hypersecretion in a murine model treated with IL-9 inhibitors [18]. Additionally IL-9 did not increase SPDEF mRNA, a key transcription factor implicated in the goblet cell hyperplasia pathway [21] whereas the IL-9/IL-13 combination and IL-13 alone showed a trend for increase in PBEC(N) and significantly increased SPDEF levels in asthmatic epithelium. Taking this data into account we feel that the lack of effect of IL-9 on goblet cell hyperplasia is real due to IL-9 demonstrating other alternative functions within our culture system.

We observed a significant reduction in the numbers of ciliated cells in PBEC(N) stimulated with IL-9, an observation which was also noted by Vermeer and colleagues [19]. Vermeer suggested the reduction in ciliated cell numbers was due to replacement with goblet cells; however our data showed that this was not the case, and that the reduction in ciliated cells is independent of goblet cell numbers. In addition, we identified no difference in total cell numbers in IL-9 stimulated cells (data not shown), consistent with inhibition of differentiation to ciliated cells, and our previous data demonstrates in this setting, greater numbers of basal, undifferentiated cells [24]. A recent study by Wadsworth *et al.* showed that in well differentiated epithelium from non-asthmatic subjects, ciliated cells expressed strong levels of matrix metalloproteinase-7 (MMP-7), which is important in the innate immunity of the airway. MMP-7 is not normally highly expressed in undifferentiated basal cells, except during wound repair [22]. In poorly differentiated epithelial cells from asthmatic patients they found MMP-7 expression to be present in basal cells, suggesting an injured and dysregulated epithelium [22]. When they stimulated normal epithelial cell lines with a Th2 cytokine mix, including IL-9 and IL-13, they observed an increased endogenous MMP-7 activity and concluded that high levels of Th2 cytokines could result in a prolonged increase in MMP-7 activity, leading to airways remodelling [22]. This would support our findings of an inhibition of differentiation to ciliated cells in PBEC(N) exposed to Th2 cytokines. Using immunofluorescence coupled with confocal microscopy we found that unstimulated PBEC(N) expressed MMP-7 at the apical surface of the epithelium, largely associated with ciliated cells, with almost no expression in the basal cells of the epithelium, as had been shown by Wadsworth *et al.* [22]. Additionally, we also showed that in PBEC(A) MMP-7 expression is less localised to the the apical surface likely due to there being fewer ciliated cells inherently in PBEC(A) [24], with higher expression also being observed throughout the basal layers of the epithelium, confirming suggestions that the asthmatic epithelium is morphologically dysregulated compared with PBEC(N) [22], [24]. We found that IL-9, IL-9/IL-13 combination and IL-13 altered the differentiation process of PBEC(N) to varying degrees, where we observed pronounced effects on the reduction of ciliated cells, the increased expression of basal epithelial cells and dysregulated localisation of MMP-7 throughout the epithelium when compared with unstimulated PBEC(N). PBEC(N) stimulated with IL-13 in particular also represented visually the unstimulated PBEC(A) epithelium. We found that MMP-7 mRNA was increased in all stimulations but not significantly, suggesting that Th2 cytokines can increase MMP-7 transcription, while also contributing to dysregulation of MMP-7 localisation in the airway combined with a reduction in ciliated cells, an observation also noted by Wadsworth *et al.* [22].

Additional effects of Th2 cytokine stimulation were observed in relation to tight junction formation. All stimulations (IL-9, IL-9/IL-13 combination and IL-13) significantly reduced the TEER values over the time course of the cultures and also highlighted significant differences between PBEC(N) and PBEC(A). However, under unstimulated conditions there were no significant differences between PBEC(N) and PBEC(A) suggesting that it is only during chronic Th2 cytokine exposure that epithelial differences between the two phenotypes become apparent. This observation has been demonstrated by our previous studies [24], [25] however another group have found significant differences between unstimulated PBEC(N) and PBEC(A) [10]. This difference may be explained by the severity of the asthmatic patients sampled. Xiao *et al.* described significant differences in TEER values between a group of severe asthmatics compared with control whereas we sampled mild asthmatic subjects for this study. Additionally, it would be interesting to see if the TEER values returned to baseline following removal of the Th2 stimulus, however that was not part of this study.

The reduction in ciliated cells and the effect the cytokines had on epithelial integrity may represent important effects *in vivo*, with resultant reduction in the tightness of the epithelium and the capacity to clear excess mucus from asthmatic airways. Recent data supports profound ciliary dysfunction associated with epithelial damage in subjects with severe asthma [30] whilst other groups have also highlighted differences in epithelial barrier function, albeit under unstimulated as well as stimulated conditions [10]. Thus, an important contribution of IL-9, in asthma, may instead be on epithelial integrity and ciliated cell number and function, leading to impaired mucus clearance, particularly in severe disease, something that can be said of IL-13 as well. This would point towards Th2 cytokines still being a therapeutic target with a focus on the effects of IL-9 on epithelial integrity, ciliated cell differentiation and mucus hypersecretion [18].

There were a number of limitations within this study. Firstly, due to the small initial cell sample we obtained from the airways of subjects we are limited in the number of experimental conditions and subsequent analyses, even following expansion in tissue culture flasks. This resulted in only being able to stimulate the cells with one concentration of IL-9. Ideally it would have been interesting to stimulate cells with a suboptimal dose of IL-13 when combined with IL-9 in addition to the dose tested to identify whether any inhibitory effects of IL-9 on IL-13 function were present. Secondly, while it would have been ideal to measure the transcription factor SPDEF in an acute fashion (ie/. hours following Th2 cytokine exposure) due to the nature of the hyopthesis and the limited number of cells, we had to analyse at the final time point, in line with all other analyses.

In conclusion, we have found that IL-9 can elicit detrimental effects on epithelial tight junctions, ciliated cell differentiation and mucus hypersecretion, rather than on goblet cell hyperplasia, as was previously thought [15], [17], [19] which may be physiologically important *in vivo*, reinforcing IL-9 as a potential therapeutic target in asthma

## Supporting Information

Primer Sequence S1 Primer Sequence S1
(DOC)
