# Supplementary material for: Chronic IL9 and IL-13 Exposure Leads to an Altered Differentiation of Ciliated Cells in a Well-Differentiated Paediatric Bronchial Epithelial Cell Model
Source: PLoS One. 2013 May 9;8(5):e61023. doi: 10.1371/journal.pone.0061023 (PMC3650011; doi:10.1371/journal.pone.0061023)
Supplement: Primer Sequence S1 — (DOC) [file pone.0061023.s001.doc]

**Primer Sequence S1**

**MUC5AC**

Forward 5’ TCC TTT CGT GTT GTC ACC GA 3’

Reverse 5’ TCT TGA TGG CCT TGG AGC 3’

**SPDEF**

Forward 5’ AGC CTA CAG AAG GGC AGT GA 3’

Reverse 5’ AAC TCA GGG GTG CAG ATG TC 3’

**MMP-7**

Forward 5’ TCT CCA TTT CCA TAG GTT G 3’

Reverse 5’ TTG ATG GGC CAG GAA ACA 3’
